# Experimental Assessment of Common Crucial Factors That Affect LoRaWAN Performance on Suburban and Rural Area Deployments

**DOI:** 10.3390/s23031316

**Published:** 2023-01-24

**Authors:** Markos Fragkopoulos, Spyridon Panagiotakis, Michail Kostakis, Evangelos K. Markakis, Nikolaos Astyrakakis, Athanasios Malamos

**Affiliations:** Department of Electrical and Computer Engineering, Hellenic Mediterranean University, 71004 Heraklion, Greece

**Keywords:** LoRaWAN, LPWAN, network planning, network administration, performance, scalability, clustering

## Abstract

LoRaWAN networks might be a technology that could facilitate extreme energy-efficient operation while offering great capacity for suburban and rural area deployment, but this can be a challenging task for a network administrator. Constraints that deform the trade-off triangle of coverage, scalability and energy efficiency need to be overcome. The scope of this study is to review the limitations of the LoRaWAN protocol in order to summarize and assess the crucial factors that affect communication performance, related to data rate allocation, bidirectional traffic and radio spectrum utilization. Based on the literature, these factors correspond mostly to configurable payload transmission parameters, including transmission interval, data rate allocation, requirement for acknowledgements and retransmission. In this work, with simulation experiments, we find that collision occurrences greatly affect channel occupancy. In particular, it was evaluated that collision occurrence is increasingly affected by transmission intervals, which have the most significant negative impact on packet delivery rate (PDR). We then validated that clustering of end nodes in the vicinity of a gateway, taking into account distance and transmission settings, can improve network scalability. This can assure distribution of the total transmission time to end nodes with respect to application-related QoS requirements. Following this clustering approach, we achieved a PDR greater than 0.90 in a simulation setting with 6000 end nodes in a 10 km coverage.

## 1. Introduction

Low-power wide-area network (LPWAN) technologies attract great attention within the academic and industry communities, due to the fact they can wirelessly connect great numbers of geographically dispersed devices at low cost. Internet of Things (IoT) deployments in suburban or rural areas could include a lot of different scenarios for various kinds of data gathering. Depending on the use case, data from sensors at remote locations could be transmitted in big- or small-sized payloads, in short or long intervals, and collected by servers with or without latency. The most important requirements for rural IoT deployments are long-range communication and large network coverage. The network should operate in places with complicated terrain where radio communications are difficult and most times even telecommunication providers are not offering coverage. At the same time, IoT networks deployed in rural areas consist of a huge number of end devices (EDs), especially when we are dealing with smart metering. The communication infrastructure should be scalable in order to connect all the EDs without affecting the performance of the whole network in a non-viable way. In addition, the required maximum lifetime for such IoT deployments may vary from 10 to 15 years, or even more. Therefore, the EDs that will be used for communication should consume minimum energy in order to prolong the lifetime of their batteries, while the total cost of each ED in terms of the annual operating cost should be low. Among these requirements, the range of communication, based on the literature, seems to be the most complicated topic. It is worth mentioning that, during the past years, the world record for the maximum distance that a LoRaWAN data packet can be successfully transmitted has been broken many times. One of these records stands at 832 km (517 miles). However, how can this record help the adoption of the protocol from a network designer? It is commonly accepted that the effective allocation of wireless resources, in order to support a big batch of devices within an area, still remains an open challenge [1]. With LoRa, we have a relatively new technology whose practical limits are not crystal clear yet. In addition, the fact that the theoretical range covers distances of the order of kilometers is creating some difficulties for real-case scenarios, both in laboratory and field tests. 

In LoRaWAN, the long range of communication is achieved by transmitting with very low data rates to improve the sensitivity of receivers within the sub-GHz industrial, scientific, and medical (ISM) radio bands. LoRaWAN in the EU offers multiple channel availability with different bandwidths within the range of 868–870 MHz in the ISM band, while the orthogonality of spreading factors (SFs) offers simultaneous transmission in the same frequency channel [2]. The protocol consists of the physical layer (LoRa) of the Open Systems Interconnection (OSI) model, which is proprietary, and a medium access and network layer, LoRaWAN, which is an open LPWAN standard. LoRaWAN was initially designed for low data-rate sensor networks, where sensors exchange packets only with the network server [3]. Within the 868 MHz band, LoRaWAN has three common 125 kHz channels by default (868.10, 868.30 and 868.50 MHz). These are the channels that the EDs are using to access the network. The network server can provide extra channels to EDs after the join process. In Europe, which is our case, 10 channels are available and used both for uplinks and downlinks. In addition, apart from LPWANs, a lot of other standardized applications also function on the same spectrum frequencies, such as RFID, alarms, wireless headphones etc. [4]. The ISM bands are license-free, thus can be utilized by almost any ED as long as it transmits based on the spectrum usage regulations. The regulation for ISM bands within Europe are different from other regions (e.g., in the US the band has no duty cycle restrictions) [5]. The usage of bandwidth is counted based on the Effective Radiated Power (ERP), while the spectrum access method for the 868 MHz ISM is based on Listen-Before-Talk (LBT) or duty cycling. The maximum ERP on LoRa basic frequencies (868.00–868.60), which are common with SigFox, is limited to 25 mW [4], although, EDs according to frequency regulation, support transmit power of up to 20 dBm (100 mW) [6]. Such power allocation is permitted for only one frequency channel (G3 band with a limit of 27 dBm), whilst 14 dBm can be used in any of six channels. The maximum transmit distance calculated with the Friis transmission equation for 14 dBm transmission power results in a theoretical range of more than 300 km [2]. For LoRa, the duty cycle calculation is based on channels with 1%, 0.1% and 10% access limit. 

Spreading factor is a customized parameter for each frame that is transmitted and is related to the chirp spread spectrum (CSS) modulation of the physical layer. The choice of SF between 7 to 12, provides a trade-off between range and throughput (data rate). By increasing the SF, the number of chips per symbol are getting increased, which reduces the signal to noise ratio (SNR) that is required for successful demodulation, but simultaneously also increases the time on air (slow transmission). Lower spreading factors increase the data transmission rate, allowing for more data to be transmitted within the ISM band’s duty cycle limits [7]. Each SF corresponds to a different data rate, which ranges from 0.3 kbps to 27 kbps [8]. 

LoRaWAN GateWays (GW) are multichannel multi-modem transceivers [5], characterized by high sensitivity due to the usage of very low data rates and sub-GHz ISM radio bands [2], that are deployed such as base stations (cellular network model) that cover large areas. In most cases LoRaWAN network operators provide connectivity as a service [8], by offering the GWs infrastructure. Any GW forwards the received messages to the associated network server for processing [9]. 

Typically, each LoRa GW supports demodulation of signals from six quasi-orthogonal logical star networks in parallel (some chips support more [10]), that corresponds to the available spreading factors [11], while transmissions are also supported on different frequency channels [5]. Specifically, the sensitivity of the GWs ranges from −130.0 dBm to −142.5 dBm depending on the spreading factor [12], while the minimum required RSSI for successful decoding requires 5 to 7 dBm more than the sensitivity for each corresponding spreading factor [9]. Therefore, when the spreading factor increases, the possibility for successfully receiving a packet on a weak link also increases, which determines a longer communication range. 

Due to the message broadcast nature of the protocol, EDs could be clustered below more than one GWs. This happens because as long as a message transmission is heard by any GW within the network, that message is considered to be received [9]. Once a GW receives a message, encapsulates its data into a UDP packet [10] and forwards it to the network server of the LoRaWAN network. Next, a JSON string is produced from the network server for forwarding, including the payload, GW information, as well as additional information for physical communication parameters, such as RSSI and SNR [1]. 

LoRaWAN deployment efficiency is mostly controlled by an adaptive data rate (ADR) mechanism which is used to control the transmission of messages between EDs and GWs [13]. It is argued that LoRaWAN networks cannot truthfully scale without using a dynamic data rate allocation scheme [14]. The optimal choice of the data rate for transmissions is the one that can ensure energy-efficient operation and increase the capacity of a single GW [14]. So, in most cases, ADR schemes aim to maximize battery life and throughput by adjusting the data rate. This method offers additional benefits on increasing the overall network capacity, as messages sent with different spreading factors are orthogonal, so they can be simultaneously received and decoded by a GW [7]. The ADR consists of two independent algorithms, one running on the ED, while the other runs on the Network Server [14].

At the moment, studies related to LoRaWAN networks are continuously taking place in order to help understanding and improving the performance of such deployments. For instance, in [9,13,15,16] it is proposed that by limiting the number of messages sent by each device per day, long power autonomy could be possible. However, this choice limits the range of suitable applications, while at the same time reduces the capacity a LoraWAN network can achieve. Such remarks indicate that an important step before a communication protocol is adopted for deployment in a wireless sensor network (WSN), is the determination of its limits for a specific application under a constrained amount of resources. However, a common problem is that during the dimensioning of a network several questions cannot be clearly answered. For example, what capacity can we achieve with three LoRaWAN GWs in a rural area of 10 square kilometers? Or, how many GWs will we need for such a terrestrial area? In addition, where do we need to place them? Where is it beneficial to place EDs when the locations of GWs are mostly pre-specified due to the ground elevations? How many devices, exchanging payloads with the server every hour or even days following a low data rate scheme, can be successfully deployed? alongway joint problems of GW placement, spreading factor assignment or power allocation are just a few issues that need to be addressed during the planning and deployment of a LoRaWAN network. Some reports [4,11,17,18] even state that the long-term performance of a LoRaWAN is not only a matter of logical and geographical layout but is also impacted by interference from other networks. A fact is that, as ISM bands become more and more congested, LoRaWAN networks may no longer provide their original performance. LoRa deployments will interfere with each other especially in close proximity scenarios [5]. Though, LoRaWAN available configuration parameters, including spreading factors, bandwidth, transmit power and duty cycling could alleviate various problems, but only when configured properly, which itself is a definite challenge.

At the moment there are several identified open issues related to LoRaWAN including network management, ADR optimization, high-density LoRaWAN installations and device interoperability [19]. Additionally, most of the existing studies are trying to tackle challenges by leveraging LoRa features to extend the capacity issue, while others designed new relaying or routing protocols or relied on signal processing techniques to cancel the interference issue [20]. conclusively, while several studies on LoRaWAN performance, scalability and security exist, the common problem of how to efficiently plan such a network has not yet received the appropriate attention [9]. At the moment, a lot of interest is taken by researchers on modeling alternative mechanisms for adaptive data rate [1,14,21], which, however, could only partially address the scalability performance of a network. 

We identify that to efficiently deploy someone a LoRaWAN network with optimal performance, he/she needs to overcome issues that deform the trade-off triangle of coverage, scalability and energy efficiency. The scope of this study is to contribute to the alleviation of the dimensioning issue.of large LoraWAN networks. To this end, we, at first, critically reviewLoRaWAN’s protocol limitations as they are revealed via the literature, and summarize the factors that affect the performance of such deployments. 

Specifically, the main issues during a LoRaWAN network design process that we identify as not properly addressed by the literature concerning the aforementioned performance trade-off could be summarized as follows:

- Estimation of the number of GWs we need to setup to cover are required network capacity within a rural area of specific size. Simultaneously, estimation of the number of EDs that can be optimally supported by a network depending on their communication parameters [9,11,13,22,23].
- Identification of the performance failures that are due to configurable parameters of LoRaWAN communication [8,13].
- Simulation-based performance evaluation depending on several scalability-related metrics such as energy consumption, PDR, throughput, coverage, and latency [19].

Through an experimental assessment we will, then, evaluate the correlation of variables such as spreading factors, GW deployment, duty cycle, transmission and interference parameters. With such experiments we attempt not only to analyze the trade-off triangle of coverage, scalability and energy efficacy, but also to dive deeper to the crucial factors that affect these communication performance indices. The result of this study is a set of guidelines towards the optimal configuration of a LoRaWAN network and the clustering of end nodes in the vicinity of a gateway, taking into account distance and transmission settings, that can improve network scalability. Appropriate simulations assess the validity of these guidelines. 

Contributions of our work include:

- Summarization of the crucial factors, as mentioned in literature, that affect communication performance of LoRaWAN networks.
- Experimental assessment of the aforementioned factors, based on insights from the literature, regarding the communication performance alterations they can cause.
- Cross-evaluation experiments that take into consideration the causalities of performance observations and the factors that affect them.
- Generation of a layered-based configuration logic, in terms of network planning guidelines, that takes into account application constraints and QoS requirements.

The goal of this work is to improve the deployment of large LoRaWAN networks by enhancing arguments for causal relationships among factors that usually are bypassed during the network design stage and contribute further the research on easy-to-use models that are required [24] to plan and assess the deployment of LoRaWAN networks. This is the challenge we aim to approach in our further studies. The structure of the paper is organized as follows: In Section 2 we review in detail how key mechanisms and concepts of LoRa technology work and how they affect the performance of such a network. Additionally, we identify and analyze the causal relationships between factors that affect performance of the network, we summarize the challenges that a LoRaWAN network needs to overcome in suburban and rural deployments, as well as the necessary metrics for argument on the following findings. Furthermore, we describe the research methodology we are going to follow, including the variables that we will control and observe, and also the experimental setup for making the corresponding measurements. In Section 3, with our controlled experiments, we contribute some new insights on coverage, scalability and energy consumption of LoRaWAN networks, which are the most important parameters for the protocol adoption. Finally, in Section 4 we discuss our findings and give our guidelines for facilitating network deployment. This proposition aims to make factor-centric observations in relation to communication performance challenges. Our approach bypasses some basic LoRAWAN concepts that network deployers are already aware of, and concentrates directly on key aspects of the technology.

## 2. Materials and Methods

### 2.1. Background

To make targeted observations we need to identify and separate, prior to our experiments, the vital factors from the trivial ones. This is a process in which we have to reduce wide problems into distinguished crucial factor complaints. Based on the literature we have identified three wide problems that are crucial for network performance. These consist of issues related to data rate allocation, bidirectional traffic and radio spectrum utilization. The next sections elaborate further on these issues.

#### 2.1.1. Capacity and Coverage Challenges Due to Radio Spectrum Issues and Collisions

LoRaWAN networks are being deployed following the cellular network model that defines network operators as providers of connectivity-as-a-service. This model is making GWs covering large areas while running applications from different vendors, which is making the co-ordination of an increased number of EDs a challenge [8]. Along with this situation, it is a fact that unplanned and uncoordinated deployment of LoRaWAN GWs, along with other LPWAN deployments accessing the same radio spectrum, could increase collisions which cause decrease in the capacity of LoRaWAN deployments. To overcome collisions in LoRa, a higher spreading factor usage is recommended to cope with higher interference levels, but this is only a partial solution because of the duty cycle time on air limits [8]. As SFs increase, the channel will be occupied for more time, increasing the possibility of collisions between packets that are concurrently transmitted even within the same network. On the other hand, because different SFs are mutually orthogonal (or pseudo-orthogonal), multiple frames can be demodulated by a GW at the same time in the same channel without collisions, as long as each one is transmitted with a different spreading factor (SF7 to SF12) [1].

It has been proved that as the number of IoT devices increases within an LPWAN, the blocking probability and probability of duty cycle violations also increase, especially in indoor cases, where there is a 5% probability of duty cycle violations for five EDs per user [15]. While the duty cycle increases the network’s robustness against interference, it also limits the downlink gateway transmissions, causing delays, which is a fact that is perceived as a general protocol limitation [19]. In detail, maximum duty cycle depends on the band, and if no LBT is used, varies from 0.1 to 10% of spectrum access [2]. This percentage is defined over 1 h and describes the maximum accumulated time that one ED can occupy a specific channel. For example, 1% allows 36 s of transmission within an hour [4]. This means that if the size of a packet is 50 bytes and the data rate is 293 bps, it needs 1.365 s to accomplish a transmission of a single packet. This implies that only 26 packets can be sent on a 1% spectrum access channel within an hour [2]. To implement these restrictions in practice, LoRaWAN specification dictates that every GW and ED ensures that any two consecutive packet transmissions are separated by at least 99 times the time-on-air of the first packet, where nothing can be transmitted [3]. To accomplish this, each ED calculates the time-on-air for each own packet in each radio channel and follows the duty cycle restrictions [2]. This workflow can be noticed if we attempt to transmit packets with very small intervals, where we will notice in most cases that a transmission was not successful due to duty cycle control and that the sub-band is blocked for a specific duration [16].

Simultaneously, it is proved that each GW can handle thousands of LoRa EDs, which means that some QoS coverage requirements are fulfilled for all six available spreading factor imaginary subnetworks corresponding to each cell area [11]. Because the LoRa physical layer is based on CSS with GFSK modulation and a high bandwidth-time product, the communication is considered somehow protected against in-band and out-band interference [25]. In addition, the GWs apply hardware signal processing techniques (I/Q inversion) when transmitting, to ensure that only EDs can hear the GW and vice versa. These techniques prevent the problem of a transmission of a GW interfering with that of an ED [3].

Nevertheless, bit error rate (BER) performance is mainly affected by possibly the same spreading factor transmissions interference, but also by background noise. While the number of EDs is increasing the amount of collisions is also rising [10]. In detail, interference exists when multiple transmissions simultaneously collide in time, frequency, and spreading factor [1]. One case exists when the increase in the number of long messages could cause higher probability of collision between packets due to the increase in the longer interval of high spreading factor messages, causing severe interferences on same or different spreading factors [6]. In addition, if there is a cluster of EDs far away from the GW, their transmissions will experience higher SNR coming from neighboring EDs [3]. The way to overcome this capacity issue on a LoRaWAN is to use multiple GWs deployment and broadcast transmissions of EDs. While a packet might not be successfully decoded by one GW, there is still a chance that it might be decoded by another [9]. In addition, an increased number of GWs increases the amount of time the network server can send downlink frames, a fact that enables ADR commands to be sent faster, which drops the average convergence time, while also offering availability for smaller spreading factors [14]. Conclusively, in order for an ED to have a successful transmission to a GW, it should be within its coverage, and there must not be any colliding packets at that GW during its transmission.

It is noted that while successful coverage of LoRa signals could be mainly attributed to noise-limited and interference-limited considerations, the reality is that the SNR threshold depicts a decreasing function of the signal-to-interference ratio (SIR) experienced by the ED of interest [11]. LoRa uses forward error correction (FEC), which allows recovery and increases reliability and range, but slightly increases the time on air because it adds some encoding overhead [3,5]. In this way there is avoidance in transmission errors due to bursts of interference. Nevertheless, the authors of [4] have provided some insights for interference within the license-free ISM bands and how it is problematic to LoRaWAN. The level of interference is caused by both radio access technologies (RFID, alarms etc. [4]) and other LPWAN deployments (LoRa and SigFox [4]). It has been measured that car keys are generating noise in the 868 MHz band. When a car is passing 5 m from the receiver it may generate noise levels up to −95 dBm. It is quoted that RFID deployment at 865–868 MHz may affect even military telecommunication devices [4]. The level of external interference such as this, in the 868 MHz EU ISM band, is anticipated to grow with the deployment of several wireless IoT solutions which are not limited to Sigfox and LoRaWAN. For that reason, it will be difficult to deploy predictable and reliable communication, with capacity and wide area coverage, due to the increased BER caused by the frequent and significant level of external interference [25]. 

#### 2.1.2. Data Rate Allocation Issues Related to Range and Power Consumption

The ADR mechanism, as mentioned before, is based on two concurrent algorithms. The one on the network side is optional but highly recommended [14] and not explicitly defined in the LoRaWAN protocol specification. Frequently, variants of this mechanism, which is not unified between deployments, are based on implementation of a simple distance-based approach in which the minimum possible transmission power and the maximum data rate that enable ED and GW communication are assigned to it. Most of the schemes that are currently available rely on EDs with static proximity (fixed locations) and on environments in which the channel attenuation is stable [7]. Semtech does provide a recommended algorithm which is quite popular for deployment and has been adopted by a lot of open source projects, such as the things network. More specifically, in Semtech’s scenario for the EU868 band, the ED decides if a default or a self-assigned data rate and transmit power should be used, or if the network server should adjust the data rate. On the side of the ED, the algorithm is based on the idea of reacting to the lack of requested downlink feedback by decreasing the data rate which results in increasing the communication range. On the network server side, if ADR is activated, it optimizes the data rate to achieve transmissions with the minimum time on air with the least possible transmission power. This data rate is calculated according to the highest SNR of the last 20 received messages. ADR is working in such a way that only the network server can increase and only the ED can decrease the data rate [10]. If the ED is not already using the highest data rate and the lowest transmission power, it calculates the most suitable data rate for this situation. Any new transmission power and data rate is communicated through the LinkADRReq MAC command within a downlink frame, and this is acknowledged with the piggybacking of a LinkADRAns MAC command in the ED’s next uplink frame [14]. If the connection is lost, the ED reduces the data rate until the message reaches the server [10].

Unfortunately, evaluations on ADR algorithms showed a failure in converge synchronization and inconsistent behavior between different implementations of the network server. The ADR algorithm on the ED is slower to converge as it has been designed to minimize the amount and maximize the flexibility of the control of plane downlink traffic, which is greatly limited by the duty-cycle regulations. The network server-side algorithm in most cases converges EDs to a data rate that can achieve greater range but is less energy -efficient. On the other hand, the ED side algorithm, which does the opposite, can potentially converge to a lossy link. The authors of [14] have found that the network convergence time is greatly increased when scaled. They proved that the Semtech ADR algorithm is effective only in stable channel conditions but not in variable conditions. In addition, they observed that the algorithm performance cannot scale due to spectrum duty-cycle regulations and that in a lossy channel the ED side algorithm convergence time is increased. Additionally, the total convergence time to reach a steady data rate for all EDs is relative to both starting data rate and also to the number of EDs within the network. Thus, the starting data rate of an ED has a huge effect on the convergence time. In contrast, network server algorithms converge to the ideal data rate much quicker than the ED side, which only changes the data rate of an ED one step at a time. The network server ADR changes begin to occur after some amount of uplink frames are received. The choice of a particular number of uplinks received to trigger changes is quite arbitrary, and it may become clear quickly that the data rate of an ED should be increased. For example, if an ED first joins the network with any data rate, this join procedure data rate becomes the first set data rate of the upcoming uplinks. Thus, when an ED joins the network, it is likely possible that it is using a much lower data rate than is actually required. Overall, in the general case, the network server-side algorithm converges to the ideal data rate for an ED more quickly. The total convergence time for EDs eventually becomes extended as the network gets bigger because of the duty-cycle regulations applied to the GW [14]. Furthermore, an evaluation on Multitech’s ADR algorithm [7] showed that when it is enabled, this causes a negative impact on the PDR, especially for EDs that are deployed far away. This scheme mainly aims to optimize throughput and dominantly assign two spreading factors, either SF12 or SF7BW250. When ADR is disabled and long-range EDs are incorrectly set up with a high data rate, which is not designed for long range, a situation occurs in which an ED can join the network but cannot effectively communicate. The joining process will adjust the spreading factor used during the joining procedure, but once the ED is joined, it will revert back to the preconfigured spreading factor. With that high data rate, it experiences very high packet loss and regularly will attempt to rejoin the network. This cycle repeats with the ED being able to join the network but not being able to transmit data successfully. In addition, note that when the link margin estimate is marginal between two assignment decisions, oscillations can occur as ADR requests are repeatedly sent, instructing the ED between different configurations [7]. Generally, if an ADR scheme is enabled, the network server transmits the LinkADRReq MAC command to control data rate (DR) and transmission power (TX) of an ED. The LinkADRReq provides only a subset of eight DR and six TX settings, which is not a utilization of the 6720 potential transmission settings combinations on the ED part [13]. Notice, that even in short coverage scenarios, the Semtech ADR is partially failed on a large scale (>1000 EDs) because it assigns on each ED the SF7 so there are quite a number of collisions [1]. Most of the current ADR approaches are nothing more than simple distance-based ED configuration mechanisms. A lot of times this fact results in non-optimal network performance, but offers an assurance within basic deployments that EDs are at least capable of communicating with GWs [9]. These remarks show how ADR convergence time and missing data can lead to loss of valuable time-on-air and drain power without reason and thus limit the overall performance on range, on capacity and on energy balance of a LoRaWAN network.

#### 2.1.3. Scalability and Energy Efficacy Issues Caused from Bidirectional Traffic

In LoRaWAN, link reliability is optionally achieved with acknowledgment (ACK) of uplink messages with a downlink [8]. In this way, the performance improves by adjusting the uplink transmission settings [15]. Once this feature is enabled, each ED waits for an ACK message after an uplink; if this message is not received, this uplink is perceived as not acknowledged and retransmitted, if retransmissions are enabled. The LoRaWAN specification recommends retransmitting up to eight times, while also recommending reducing the data rate between every two unsuccessful transmission attempts in order to achieve more robust connectivity [3]. If the number of retries is set to zero and ACK is not received, the packet is not automatically retransmitted and the send functions indicate failure [7]. LoRa unacknowledged configuration defines transmissions according to the worst link budget by using the lowest data rate since there is no feedback. Such configuration leads to a long time on air and high collision rates [15]. To ensure network scalability, it is a common practice that LoRaWAN EDs are set to minimize the use of ACKs. Apart from the feedback of receiving a message, EDs are also able to periodically validate the link quality by checking if the link still exists. In this case, the ED requests a link check message and a GW will respond, which indicates that the ED is still connected [7]. If the link quality is good, it could be possible to transmit with a higher data rate and lower transmission power. Each application should specify the number of link check failures that indicate that network connection is lost and how often these checks should be applied [7].

At the moment, it is quite common for studies to report optimistic achievements regarding LoRaWAN network capacity. On the contrary, some findings are reported that conclude that introduction of downlink traffic can have a significant impact on the uplink throughput [3]. There are a number of required and optional functions that use downlink messages apart from just data transmitted from the application. A downlink message is coming in response or prior to an uplink from an ED on the following cases: acknowledgement of confirmed uplink packets; reporting of uplink quality for EDs; adapting communication parameters for uplink transmissions; settings for RX2 window and offset between uplink and RX1 window; setting transmission duty cycle for EDs; requesting battery status and quality of downlink from EDs; creation or modification of settings of the channels to be used for communication; and setting the time offset between the uplink TX and the RX1 window. Some findings showed that the GW duty cycle limitations, and also the collisions, are introducing an unreliable downlink delivery. There is a huge impact on this when the network scales, especially in relation with the ACKs. For Class A EDs, the GW transmits any downlink messages and ACKs in the two receive windows (RX1 and RX2). By default, in Europe, window two is fixed to use 869.525 MHz and SF12. In this case, the channel is occupied for a very long time if the GW replies to EDs [5]. In addition, if the GW must transmit an ACK it is unable to receive other packets simultaneously [7], while there is also a delay window between the receipt of a message from the GW and the first receive window of the sender ED opens [5]. Apart from these, as the network size increases, the GW is required to transmit more downlink traffic, exhausting its regulatory time on air allocation more often. Each time this happens, the GW gets blocked for some time and cannot transmit. For a network of 5000 EDs, successful downlink transmissions, with ACKs enabled, drops to about 35% while without ACKs it dropped to 60%. So, there is evidence that as the percentage of uplink messages requiring an ACK increases, the network performance degrades, which means that the network cannot scale in such a case. In addition, the blocking of GW opportunities to transmit due to duty cycle limitation causes an increase to the number of retransmissions, leading to a huge increase in the energy consumption on EDs, which is about 500% for the 100% packet acknowledgement scenario. This happens because, when EDs do not hear back from a GW, they assume that their uplink has lost due to the lack of confirmation. Furthermore, apart from the battery drain, retransmissions introduce a large drop in network goodput, especially on larger network sizes. Additionally, the introduction of downlinks in addition to the ACKs leads to competition between them, thus retransmissions increase, and end up reducing the uplink goodput even further [3]. In summary, deployment parameters are crucial for the overall energy efficiency of a network [26].

#### 2.1.4. Communication Performance Metrics

Link quality is characterized by received signal strength indicator (RSSI), but varies greatly among chipset manufacturers [27]. When measured on a logarithmic scale, in decibels (dB) or dBm, values closer to zero are indicating a better signal [16]. RSSI is sometimes used during evaluations to characterize performance as a function of distance or environmental conditions [1], while within LoRa, its value from the messages that arrive at the GW is used for the selection of a specific spreading factor [1]. Generally, existing work has shown that a high RSSI can be used as an indicator of a very good link [13] and depends on the distance between the GW and the ED, according to a path loss model [1]. However, in a lot of cases RSSI does not correspond linearly with the distance between receiver and transmitter. SNR is a key factor to evaluate and analyze the overall performance of the network and can have a value between −20 dB and 20 dB, under the highest spreading factor (SF12) or under optimum environment, respectively [27]. On evaluation there are some other metrics that are also used. Packet error rate (PER) or PDR are used as scalability performance metrics where PER is defined as the rate of the packet not successfully received by the GWs, while PDR is the probability that an ED has a successful transmission to the GW. A scalable technology is one that provides low PER or high PDR. Another metric that counts the average number of packets transmitted successfully by an ED is energy efficacy (EE), which is a performance metric for power consumption and measured for every 1 unit of transmission energy. A serious factor that affects power consumption is the packet transmission time which is measured by time on air (ToA) which is the total time between ED transmission and GW reception. Higher ToA is influenced by the spreading factor of the transmission and the size of the payload [27]. Because LoRaWAN uses link adaptation, it is shown that ToA varies from 22 ms to 860 ms depending on the coupling loss [25]. Furthermore, to measure the overall network behavior and scalability, and not for each ED independently, the data extraction rate (DER) is used, which is computed in a given time period, and represents the ratio between messages that have correctly been received by all the GWs and the transmitted messages from all EDs [1].

### 2.2. Crucial Factors Summarization and Experimental Methodology

As LoRaWAN is using the license-free ISM bands, one can think of that as a huge advantage. If we analyze it from the scope of fixed costs, it is. On the other hand, when we are planning massive-capacity IoT networks in suburban and rural areas within Europe we are also facing problems that arise from the ISM spectrum access regulations. The duty cycle limitations revealed a lot of issues that are common for the majority of large- scale deployments. To overcome this issue the obvious answer is to set communication parameters in a way to achieve balance between capacity and coverage without reaching the duty cycle limits. Some studies have proven that in this step of the deployment several other problems could occur related to collisions, interference and power consumption of the EDs. As we mentioned before, both the ADR mechanism and bidirectional traffic issues are pointing somehow to these spectrum access regulations as a factor that exponentially affects the network performance in a non-recovery way. ADR mechanism convergence time is increasing as DR is increasing. Convergence time indirectly affects the ToA, which is increased directly when SF or payload size increases. Additionally, we have seen that bidirectional traffic causes issues of scalability, especially because downlinks in Europe are transmitted with SF12 which results in long transmission ToA. The impact is that as the number of EDs asking for ACKs increased, the successfully transmitted downlinks decreased, while as the number of retransmissions of EDs decreased the acknowledged uplinks decreased.

The main issue is that all variables between uplinks that were acknowledged, successfully transmitted downlinks and convergence time are indirectly negatively affected by the increase of the number of deployed EDs, which drives us to the conclusion that every crucial factor tends to reflect on scalability. Simultaneously, a frequent and significant level of external interference is also argued as causing issues that lead to the limitation of LoRaWAN deployment capacity. This is a reasoning that undermines the optimal planning of the network itself even if scalability issues have been solved. AIt is drives us to the restriction of the total network capacity.

Our methodology will be led by previous experiments in several literature evaluations. To facilitate the LoRaWAN community with some new insights about the utilization of worthwhile network deployment recipes we will try to evaluate different configuration scenarios to export the crucial communication factors’ proximity. To claim anything on these we have to make a lot of observations on the cross relation of different factors. These observations will be led by the exploration of data rate allocation methodologies without ADR and SF12 and ACK avoidance, in an effort to break down these issues into simpler ones. The observations need to be made in a series of cases in a cause-to-response manner. We are defining our significant inputs (controlled variables) for this study (Table 1) among the DR, TX, distance of ED from GW, GW number, payload size, transmission interval, retransmissions, ACKs and the number of EDs. The goal is to study the relationships and causalities of these independent variables in order to create some directions for the network design process. These directions must be in a way that the aforementioned application-oriented QoS demands are respected.

The fact that commercial network deployments can include thousands of EDs force us to make some observations in a simulator-based manner. This method offers us the ability to stress-test the network while at the same time we can evaluate numerous different parametrizations per ED. In this way the derivation of observations about scalability limits of the protocol, which seems to be the most important, will be facilitated.

**Figure 1:**
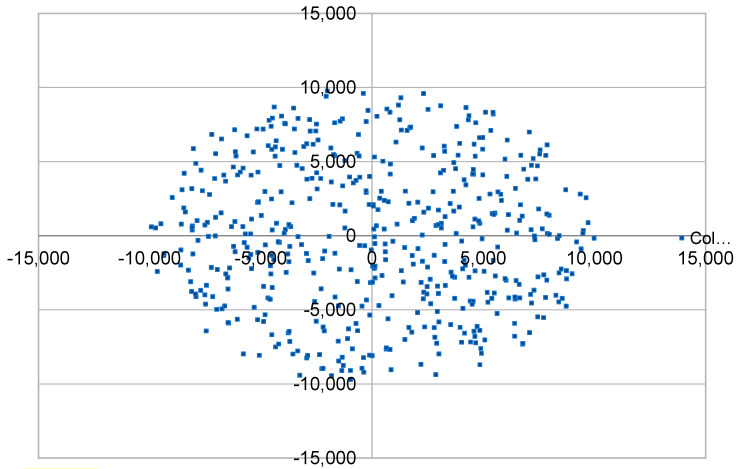
Simulation scenario with disc end node spatial distribution.

**Figure 2:**
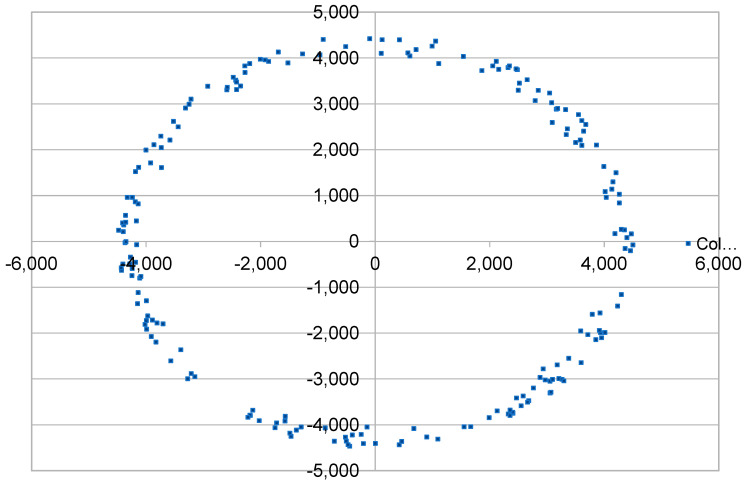
Simulation scenario with disc periphery end node spatial distribution.

**Figure 3:**
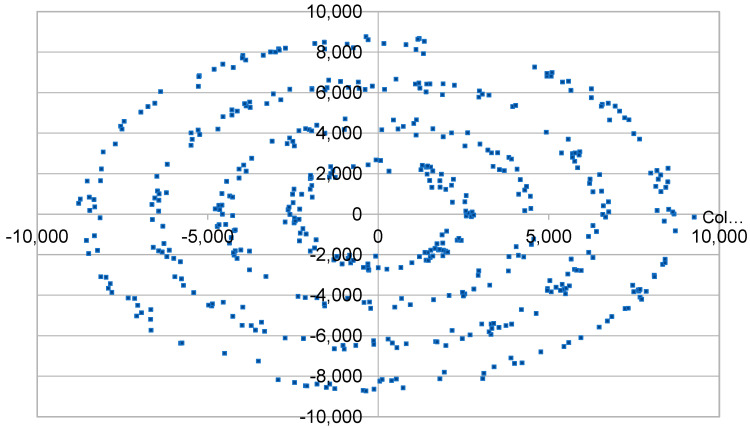
Simulation scenario with multiple rings end node spatial distribution.

The ns3 simulator is being used with the addition of the LoRaWAN module. Previous experiments that were reviewed in the literature—which also utilize this software—seem from the insights to be very powerful. To accomplish our goals we need to test LoRaWAN limits by developing some measurement setups based on Table 1. Several repeats of the tests need to be made for every factor as well as for every level of each factor. We will also randomly make observations between the different setups on different variables to enrich our statistical approach. In each simulation scenario a LoRaWAN network is created in an environment with modeled congestion to evaluate each time the possibility of message loss. Specifically, the height of the GW antenna has been set to 15 m, the height of the ED antenna to 2 m and the path loss exponent to 3.76. A crowd of EDs is created and placed with random distribution within a disc (Figure 1) of a specific radius or in the periphery of such discs (Figure 2) or even in disc rings (Figure 3), depending on the scenario under consideration. As of this study, path loss staticness and ED distribution randomness, but also external interference and downlinks absence are perceived as the uncontrolled variables that can cause false correlations or improper analysis of the results. Then, we are assigning values for the parameters SF, TX as well as whether the messages that will be sent require acknowledgement, and if so, which is the maximum number of retransmissions. In addition, GWs are placed in selected spatial positions. Finally, the size of the payload and the intermediate period of creation and sending messages from each ED (uplink) as well as the total simulation time of the test are noted. ADR will be disabled in all cases because in some cases it caused more issues than it solved [28]. It is found that while ADR decides to update SF for a node based on channel conditions, it does not consider the impact of collisions resulting from the increasing number of nodes within the same network [29] to be able to manually control parameters independently. Within our study, important insights are expected regarding the impact of the data rate allocation of the EDs. Thus, we need to manually control parameters such as this independently in order to make causality assessments, especially for spreading factors. EDs are class A, while in the vast majority of cases we consider that all EDs are creating messages of common size and transmission interval. For the implementation we developed a simulation program for the LoRaWAN module with the ability to cluster EDs that differ, in their distance from the GW, in transmission parameters (ACK, retransmissions, TX, DR) and in the size of the transmitted package and the interval between successive transmission needs. In addition, some bash scripts were developed for the dynamic modification of the relevant parameters and the repetitive execution of simulations. Experimental assessment of downlinks impact have stayed out of the scope of the simulations because there is no such implementation on the module. The only way we have determined some relevant impact is the acknowledgement scenario in which downlinks are also utilized. External interference has also been bypassed throughout the experiments for the same reason.

We will use packet delivery ratio (PDR) to make our assessment and to argue on the causality between observations on the communication performance of LoRaWAN. Our experiments include four different dependent variables. These variables are expressing different message transmission failure reasons. All of these dependent variables affect the overall network performance, which is measured in PDR, which is calculated from the total received packets divided by the total packets sent. In our simulations the packets that are not received successfully by at least one GW could have been affected by several circumstances. Interference, sensitivity issues, inability to find a free channel on a GW, or the GW is busy while transceiving a message. Interference is the most common failure reason for collisions while scaling a network. Interference is dependent on the SNR of the transmission. Sensitivity failure is directly related to the distance of a transmission and is dependent on the signal RSSI. In summary, failed transmissions are separated in the following four groups:

- Sensitivity: Related to distance and obstacles and is depended mostly on TX power and distance;
- Interference: Related to radio signal interference between simultaneous transmission of the network or even from other networks on the same radio frequencies spectrum. Interference within the same network causing collisions that are indirectly caused from configurations such as data rate, EDs number, TX power, ACKs, retries or generally by ToA (transmission interval and payload size);
- GW channel availability: Occurs when all concentrators of a GW that are listening to a channel are occupied for an active transmission. This is directly related to the number of EDs, the retransmissions and ToA;
- GW in TX state: Occurs when GW is busy by transceiving a downlink. This depended mostly on ACKs but also in application logic that handles downlink processes.

Throughout our literature review we have identified a vast number of cross-relations between factors that we need to experimentally assess. Thus, our experimentation was planned based on these relations that we knew beforehand. Firstly, in all radio communications, PDR is clearly related to the range. In LoRaWAN specifically, according to our survey, which we have analysed in the background chapter, PDR is also decreased proportional to the amount of EDs that are communicating with a GW—while simultaneously SFs or ACKs or retransmissions or payload size also increased. It is important to mention that, based on previous studies, deploying more GWs can overcome these causalities [24] but also cause some scalability issues when more than one GW is accessible by an ED. Furthermore, if the ToA of a transmission between an ED and a GW increases, then the power consumption will also increase. Apart from ToA, power consumption of EDs is related inversely to the interval between transmissions and to the DR, and is directly affected by the TX parameter of the ED which controls the transmission power. If TX is decreased, the range of the communication is also decreased, which is also happening if the SF is decreased. Finally, capacity is uniformly increased if the channels to be used for the communication also increase, which indirectly leads to more capacity on the long ED clusters, which implies better coverage.

## 3. Results & Observations

After experimenting with hundreds of different deployments on the simulator we have extracted some cause-to-response observations regarding nearly every factor that can harm a deployment. The experiments have been repeated several times for each setup in order to achieve statistical accuracy. However, it should be noted that, when repeating the same experiments, the results were exactly the same—thus the standard deviation between the results was 0.

### 3.1. Capacity of Spreading Factors Clusters

**Figure 4:**
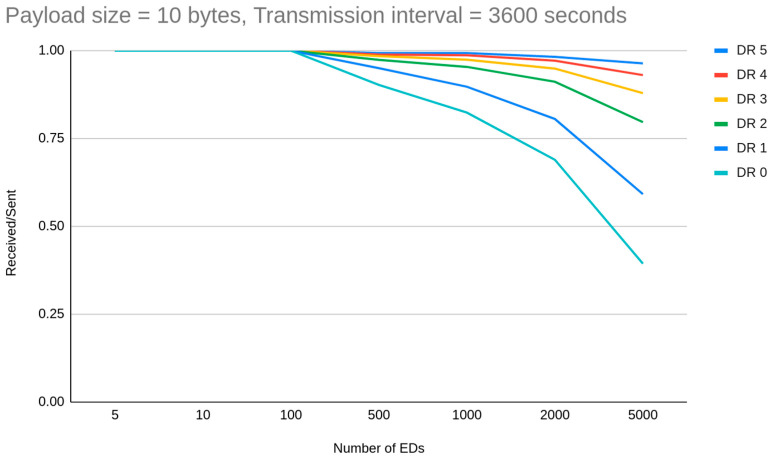
How DR allocation affects network capacity.

The first thing a LoRaWAN deployer has in mind is that different spreading factors are directly related only to the range of the communication. Instead, spreading factor allocation is argued widely as an essential factor for the reduction of interference between node transmissions, due to the demodulation process. In general cases when the number of EDs is increased above 100 the PDR is decreased. Apart from certain observations, we have to mention that when we compared the total simulations where PDR exceed or is equal to 0.95, we notice that for experiments from 300 to 1000 EDs, 16 simulations out of 77 were on that level, while for simulations from 80 to 100 EDs, 30 out of 53 simulations were on that level. Specifically, we have noticed for example that for a deployment with 10 min transmission intervals for 1000 Eds, the PDR due to collisions drops from 0.90 for DR5 to 0.10 for DR0. This happens because when the number of EDs increases the possibility for interference increases as per the ToA which is SF-related. The same is also performed in high SFs for channel availability. So, we getting more arguments for assumption that as SF increases, fewer EDs on the network can be set to this. Thus, schematically, we can tentatively claim that each transmission interval has a corresponding maximum supported number of EDs, different for each DR cluster. In simulations with a setup of 1 h transmission interval, the capacity of EDs when the goal is to achieve more than 0.90 PDR is greatly reduced based on the DR allocation (Figure 4). DR4 and DR5 clusters showed that they can handle successful transmissions for 5000 or more EDs respectively, DR3 around 3000 EDs, DR2 1500 EDs, DR1 nearly 500 EDs and DR0 around 200 EDs. On the other hand, we have noticed that when the distance is increased the ToA is increased which leads to slight PDR reduction caused by interference.

**Figure 5:**
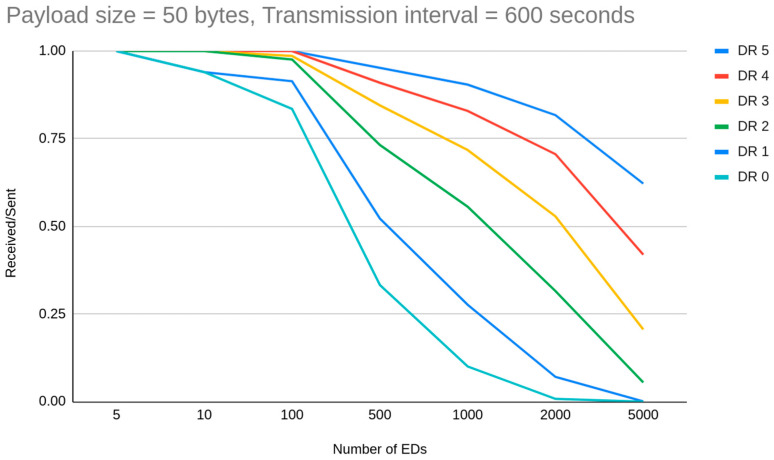
DR allocation can further downgrade the capacity by decreasing transmission interval and increasing payload size.

On the other hand, if we increase the frequency of transmissions, we can see that DR cluster capacity is degraded significantly. We are seeing this impact on simulations with a 10 min transmission interval and 50 byte payloads (Figure 5). The corresponding DR cluster for this case drops to around 800 for DR5, 600 for DR4, 400 for DR3, 200 for DR2, 100 for DR1 and below 20 for DR0.

### 3.2. Size of Payload & Transmission Interval

Duty cycle limitations are not as important as we thought prior to our experiments. We perceived these regulations as a great obstacle for the network scalability, but on the other hand a lot of preceding factors affect this aspect of LPWAN deployments. Some experiments with 1000 EDs, distributed in every DR spectrum based on distance, reveal to us the spectrum limits in terms of data acquisition frequency. We have noticed that while 100% of 10 byte payloads are sent with a 200 s transmission interval, 89% were sent with a 100 s interval and 71% with a 60 s interval due to reaching the duty cycle limitation. The fact is that such transmission interval settings are, in one way or another, not viable for large scale deployments as we will argue with the following observations.

**Figure 6:**
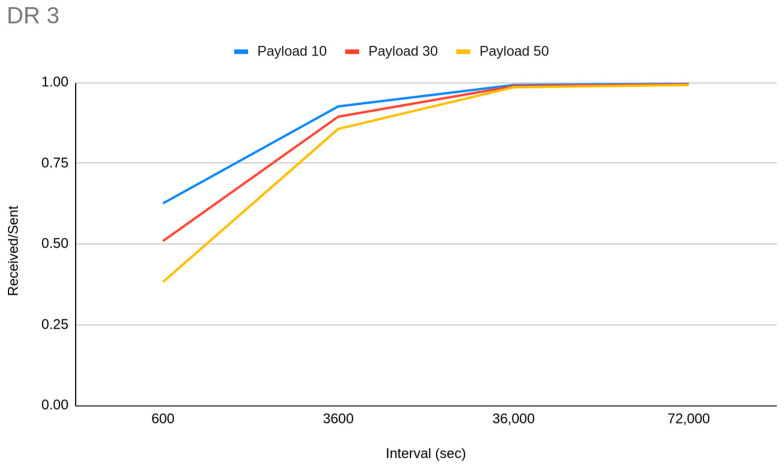
PDR differentiation derived from payload size and transmission interval relation.

For 5000 EDs we can achieve 0.96 PDR when we have small payload (10 bytes), medium transmission interval (1 h), in a range of 2 km with all the devices set to DR5 to avoid long ToA. With the same settings, but using DR3 devices instead, the PDR reduced to 0.88 and the higher chance of successful packet decoding due to the higher SF is not real. We can therefore determine that the importance of the ToA factor is higher than SF ability to decode in noisy environments, when we analyze collisions. It is worth mentioning that on a series of simulations where we have set a very short transmission interval (1 min), the PDR cannot climb higher than 0.11 when more than 2000 EDs exist on the network, and is zero for lower DRs, with even two GWs in some cases. Such low PDR in that case was caused due to GW channel unavailability. Because the distance is some km, the transmission gets wider in time so a huge number of messages arrive at the same time to the GW. Even a random transmission time within the range of 60 s, which is the time between two consecutive payloads, is not enough. The only way to have channel availability in such a small transmission interval is to use DR5 and deploy less than 2000 EDs, but again the collision problem due to ToA is huge (0.54 PDR). From the gateway prespective, based on our experiments, for less than 100 EDs, GW channels are enough in any case, but the GW may be unavailable for receiving packets when the transmission interval is less than 10 minutes or when the transmission retries are getting increased. When we reduce the EDs to 3000, with the previous settings, PDR jumps to 0.98, but reduces if we differentiate the payload size (0.97 for 30 bytes, 0.95 for 50 bytes). When we have a lot of EDs (3000) and high coverage is required, the only way to reduce the collisions with increased ToA (only DR0 and 50 byte payload size) is to increase the transmission interval. In such a way we can achieve 0.95 and 0.97 PDR for 50 bytes and 10 bytes respectively. It needs to be noted that, for such rare in time transmission interval settings (>10 h), the PDR is 1.0 when the DR is 4 or 5—independentently of the payload size—and drops to 0.99 when DR3 is used. Another way to decrease the collisions, when you have a lot of EDs in a great distance that need to be allocated in DR0, is to deploy more gateways. Our observation here is that for a network of 400 EDs with a 10 min interval between the transmissions of 20 byte payloads, the PDR is 0.74 when four GWs are deployed, while with one GW it is 0.56, but the deployment of more EDs along with more GWs is not a choice at all to scale the network. This observation reveals that if we want to deploy a medium-to-small capacity network (<500 EDs), with great coverage and kind of frequent data acquisition the only way is to deploy two GWs instead of one. This situation is not analogous for more EDs. In another experiment with 1000 EDs in DR3 we have noticed that if we send the same amount of data in one payload in a more rare interval it is better than sending every time data are available. The main impact is caused by the overhead each different payload has, which increases the overall ToA. In detail, we have achieved a PDR of 0.96 when sending 50 byte payloads in 50 s interval, 0.93 when sending 20 byte payloads every 20 min and 0.88 by sending 10 byte payloads in 10 min interval. Figure 6 demonstrates the PDR differentiation which is derived from payload size and transmission interval relation. It is worth mentioning that for medium-sized or larger networks, the GW starts to run out of available channels if the transmission interval is set to 1 min or less.

**Figure 7:**
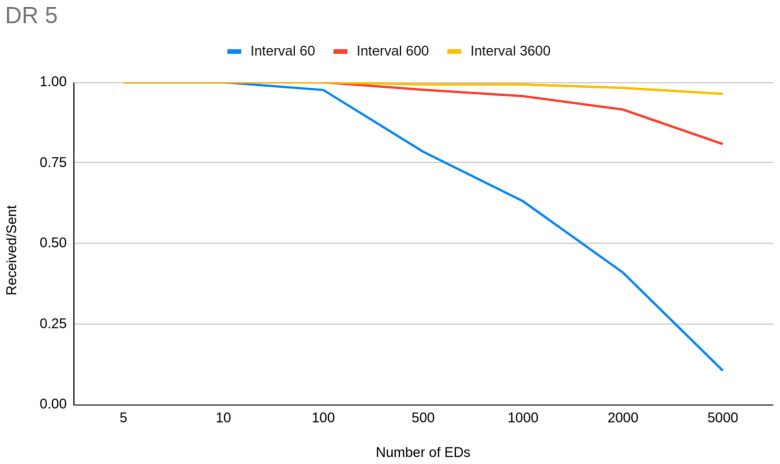
DR5 capacity related to transmission interval.

Those observations imply that when we want to scale our network and have a capacity of several thousand EDs with medium coverage, devices should be clustered into groups of data acquisition frequency. End devices that have to report data rarely could be set to a lower DR setting while the EDs from those from which we need data more often should be set to a higher DR setting, with the premise of exhausting the 50 byte size limit in each payload. Nevertheless, the impact that short transmission intervals have on PDR and thus on network capacity is so crucial that even in DR5 there are limits. We can see that when transmission is very frequent, even with 10 byte payloads, the capacity for DR5 also drops to around 100 EDs (Figure 7). It is obvious that for the avoidance of interference, transmission interval is a much more important parameter than higher spreading factor distribution.

**Figure 8:**
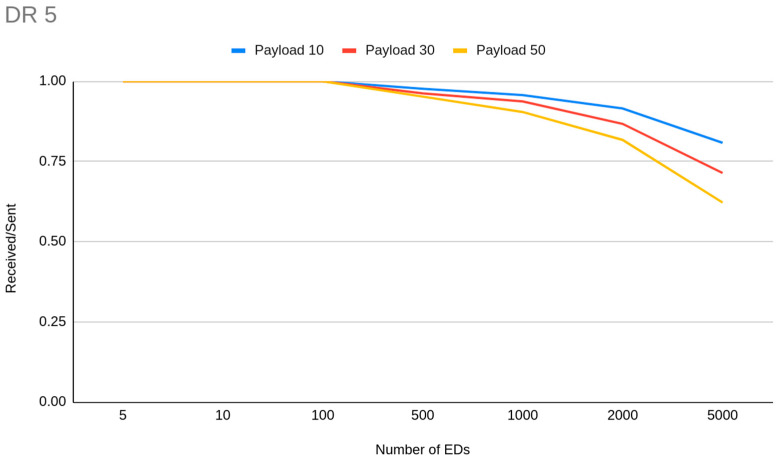
PDR differentiation derived from payload size and number of EDs relation.

In general, we can summarize that if there is a certain amount of data to be sent it is better to send them in one big payload in more rare intervals, than to send data when they are available with shorter payloads. This is not the perfect choice, but one with the least possible drawbacks related to transmission interval context issues (Figure 8).

In addition, it is proven that transmission intervals less than 600 s can exponentially harm network performance, when there are more than 100 devices or when a lot of devices use lower data rates. We are claiming that a DR-based distance relative transmission interval distribution method will be vital to scale a network, but this is only applicable if we can break down the EDs, within the application layer, in different classes of data acquisition frequency.

### 3.3. Acknowledgements, Retransmissions and Gateway Availability

The ACK payload with one retransmission has been shown to work satisfactorily (PDR is 0.95) for deployment of 80 EDs with transmission interval of 10 min and 50 byte payloads. The impact of the ACKs on the gateway side is also active here, independently of the small number of EDs. The gateway is still unable to hear five out of 100 messages, which is the main issue compared to collisions. The aforementioned simulation has been set up in three clusters of nodes, distributed at 1000 m or less, with DR3 to DR5 depending on the distance. We have also tried to scale the above to a 10-times bigger size network that, with the same settings, saw its performance drop to 0.87 PDR. In a parallel simulation we have moved more than half of the devices to DR0 and the PDR has been differentiated a lot and rose to 0.77 and cannot reach more than 0.80 even with one retry. Surely, if we set the transmission interval to 1 h then the PDR is even better than without the DR0 EDs (0.96), which again argues on the importance of rare transmissions for any case. Instead, if we keep the transmission interval the same and reduce the packet size to 10 bytes the PDR increase is not the same (0.84). Based on our analysis, the observation that when we import DR0 EDs on short data acquisition frequency deployments, PDR reduced a lot, occurred in a stepwise fashion. When GW is acknowledging payloads, which are downlinks, in SF12, suddenly a lot more traffic on the same SF occurred, creating a lot more interference. This fact increases the retransmissions on the same SF, which increases the overall traffic that the GW must handle, with the extra drawback that this kind of traffic occupies the channel for a long time. This sequence causes a high transmission failure rate due to the busy condition of the GW. This drawback is always the case when we use ACKs on a medium- or larger-sized network that has DR0 devices in Europe, where SF12 transceiving happens in the same SF as the GW acknowledges the payload receiving success to all nodes. To prove this, we have conducted some targeted simulations with 300 EDs within a 3 km axis, with the goal of getting the most out of the retransmission LoRaWAN feature. We have achieved 0.99 PDR when configuring all EDs to DR5, two retries and a 10 min transmission interval, that falls to 0.97 when all EDs were set to DR3 (due to increased collisions due to ToA). As expected, when we deploy the same network, but with the EDs set to DR0, the PDR rose to 0.58 and even when we increased the interval between transmissions to around 17 min, the PDR was 0.74. In an attempt to scale a network with ACK, we simulated 3000 EDs to transmit every 1 h a short message on DR3 and the results were really satisfying, reaching 0.95 PDR. Achieving as high a PDR as before (0.95) without ACK, with exactly the same settings, can be achieved if we reduce the EDs to 2000.

Simultaneously, we can argue here that deployments with medium capacity that require high data acquisition frequency, wide range and acknowledged transmissions can perform (PDR > 0.75) only if more than one GW has been deployed to the network, but we notice that if we use four instead of two gateways, PDR increased only 2%, so it is a useless choice on its own. On the contrary, if we deploy more GWs on a small-capacity network without ACK we can see this is a useless choice because no difference to the PDR is observed. So, applications that need to achieve high QoS, which depends on messages ACK or/and applications that need a lot of downlinks, need to deploy more gateways. This is a necessity because each ED on a network could have a GW fail when trying to listen on an uplink due to being in TX condition (transceiving). In our experiments we noticed that the messages that were not delivered successfully due to GW TX condition, when only one GW is deployed, are about 5–15% of the total transmissions and their number is based on the network size (100 to 3000 EDs respectively).

### 3.4. Transmission Power Allocation and Relation to Distance

**Figure 9:**
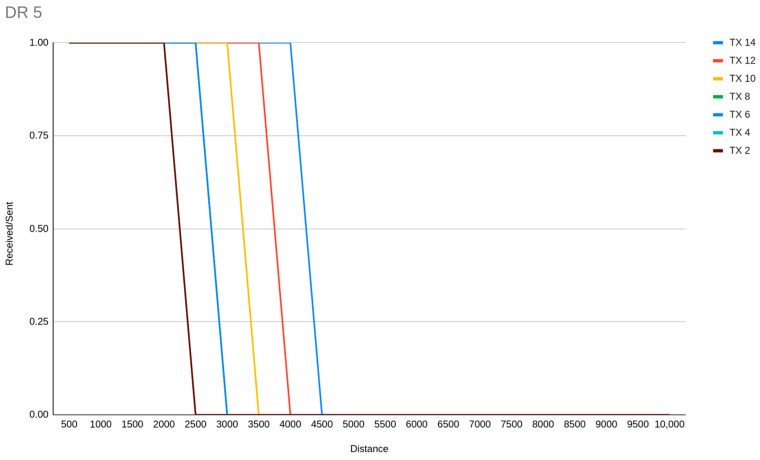
DR5 range of communication with different TX power.

We have conducted some line-of-sight experiment simulations related to range, DR allocation and TX power. Our observation is that TX and SF are inversely proportional regarding the impact of communication range. For example, an ED link of 4 km distance could be achieved by both DR4 with TX 10 and DR3 with TX 6. Additionally, for TX power levels, our experiments showed that sensitivity fails may occur when a transmission is weak to travel a wide distance. The highlight of TX power centric experiments is the discovery of the abilities of DR5 in terms of range, which could be from around 2 km or less with TX 2, to a maximum of around 4 km with TX 14 (Figure 9).

**Figure 10:**
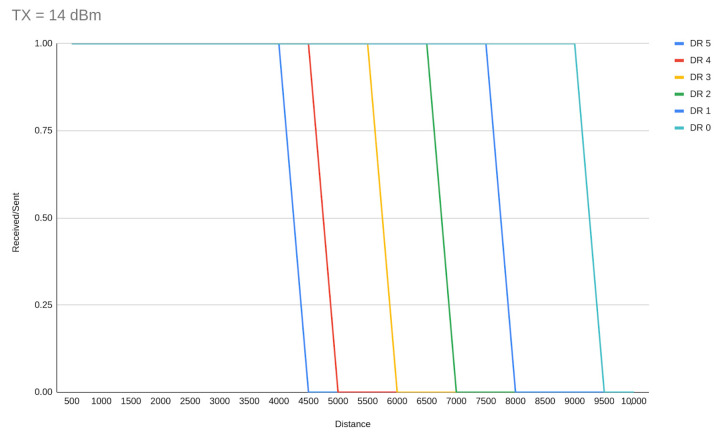
Range of communication with TX power of 14 dBm.

**Figure 11:**
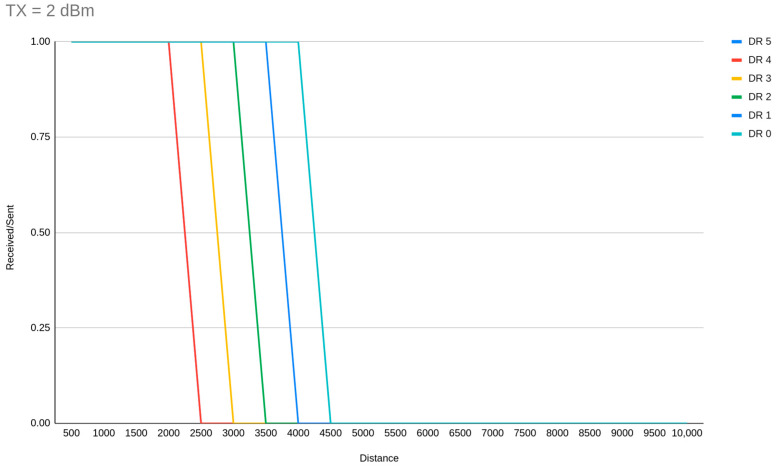
Range of communication with TX power of 2 dBm.

A derivative from that is that DR5 must be allocated to the majority of the EDs on a network, with TX distribution to achieve less power consumption wherever is possible. In a lot of studies, SF7 allocation (DR5) is reported as an option that monopolizes the nodes where ADR is enabled. With our experiments we can conclude that DR5 is the best choice for medium-distance deployments (<4 km) because it achieves the shortest ToA which is directly related to interference, due to less possible channel occupation, and also to power consumption. For these reasons, it should be used in a higher ratio among other DRs and in wider range network deployments. Figure 10 and Figure 11 demonstrate the DR range limits (as per the simulator implementation) for TX = 14 dBm and 2 dBm respectively.

An additional conclusion that has been derived from our distance-based simulations is that as the SF increases the TX should be smaller in order to achieve common maintenance time between the EDs. If this does not happen, EDs with higher SF will need bigger batteries. This can be shown by the fact that, when the spreading factor is increased, the possibility of successfully receiving a packet on a weak link is also increased.

## 4. Discussion

Based on our observations we can argue that collisions (interference between different transmissions within the same network channel) have the most significant negative impact on PDR. We have explored numerous possibilities and we have seen conclusively that ToA is directly related to collisions. Specifically, the bigger the SF, the payload size and the distance, the longer a transmission would last. Further, we can say that the longer a spectrum channel is occupied, the higher the possibility of collisions. This means that we do not care about the ToA of a payload transmission itself but the sum of its repetitions, acknowledgments or retries of all EDs that occurred within the same SF. Thus, we can say that the most crucial factors that affect LoRaWAN performance are those that affect the channel occupancy exponentially. Based on these observations, we determine that the number of EDs that are deployed and the transmission interval are the most important factors. However, the more direct aspect of the problem is not the overall number of EDs but the number of them within each SF, especially as the SF gets higher. It is clear that SF7 can handle a tremendous number of EDs itself but cannot work on its own if we want wider coverage. Finally, we can claim that it is far better to use more GWs to avoid the usage of SF12 completely, which causes a huge number of problems for realistic data acquisition frequency requirements, especially when ACKs are active because in Europe most deployments use SF12 for RX2.

### 4.1. Safe Pathways to Deploy a LoRaWAN Network

After experimentation with different variables based on the influence of crucial factors, we are proposing some optimal deployments aiming to include as many EDs as possible (scalability), cover a wide range (coverage) and use as little power as possible (power efficiency). In simple words, the parameters for the protocol adoption is the trade-off between scalability, coverage and power consumption and a parallel goal is to offer LoRaWAN-wide solutions depending on the application requirements, in order to avoid the withdrawal to other LPWAN alternatives (NB-IoT, Sigfox) without reason [30].

For small-scale networks (<100 EDs), which is the easiest situation, it is safe to deploy EDs to only DR5 when the required coverage within a rural area is under 3–4 km. In this case the data acquisition frequency could be even less than a minute. When we want to reach medium coverage on such a deployment we can additionally deploy EDs on DR4, DR3, DR2, DR1, but the transmission interval needs to be increased in most cases. For reaching the maximum coverage on small networks we can deploy DR0 EDs but only for transmitting in intervals more than 1 h. Once the capacity increases (<500 EDS) and the required range is medium (<6 Km) the network can work flawlessly when data acquisition has been set to 10 min or more and the data rates used are distributed to DR3 or greater. Once the network gets bigger the safe option is to also decrease the data acquisition frequency. It is proved that you can have the highest data acquisition assurance once on a medium-to-large network (<1000 EDS) the data acquisition frequency drops to 1 h, while only DR4 and DR5 EDs are deployed for short-to-medium (<4 km) coverage deployments, that involve DR2 and DR3 along with ACKs for medium-to-large (<6 km) coverage deployments. Data acquisition frequency of 1 h can certainly support even large-capacity deployments (<5000 EDs). For such dimensioned networks only DR5 should be used for short-to-medium (<4 km) communication range requirements, or DR2 to DR5 with ACKs for medium coverage ones (<6 km). When large capacity and wide range are both requirements for a network then the way to have the highest possible acquisition assurance is to set the transmission interval to 12 h, deploy EDs in all DRs (DR0 for less than 20% of EDs) and activate ACKs with one retransmission.

It is worth mentioning here that The Things Industries proposed and facilitated some great concepts to overcome a lot of the issues we have also identified. A fair access policy and the ability to configure RX2 to SF9 are by far the most advanced methods to maintain great capacity common infrastructure LoRaWAN networks while respecting the shared ISM spectrum.

### 4.2. Proposed Approach for LoRaWAN Network Deployment

When we can configure a LoRaWAN network of thousands of devices over a range of several km with the least power consumption, we can define some different application-oriented demands—namely data acquisition assurance, data acquisition frequency, and data size—which all are different aspects of Quality of service (QoS), as long as a hybrid solution for serving applications based on data-level requirements such as the distinction of data and so in EDs level QoS. The level of data acquisition assurance is not directly connected with ACK and retransmissions, as it could be, but to the PDR cut-off which is acceptable for our needs. For example, this cut-off could be 0.50 if our application is not precise centric and we do not care if we lose one out of two messages.

By following the below application-wide clustering approach method, we have gathered some great insights. We have achieved greater than 0.90 PDR on a large network (6000 EDs) on a wide coverage (10 km) with a data acquisition frequency range from 10 min to 12 h and medium to very high data acquisition assurance.

The proposed clustering approach is as follows:

1. Count the overall EDs that are needed;
2. Deploy as many GW as are needed to avoid as much as possible the DR0 allocation that could be necessary for distantly deployed nodes. Do not exceed 900 nodes per GW channel [31].
3. Separate the EDs into several clusters based on the distance from the closest gateway. The clusters should be in donut shape with the inner cycle being the shortest distance away and the outer being the furthest;
4. If there is not an application-wide QoS level, further distribute the EDs in each distance cluster to several new clusters based on QoS aspects (data acquisition assurance, data acquisition frequency and data size);
5. Distribute the EDs for each of the previous clusters to DR-TX groups based on the following criteria: (a) The larger the distance and data acquisition frequency, the larger the DR that should be allocated, while being proportional to the acknowledgment and amount of retransmissions, (b) The higher the DR, the higher the TX power needed to simultaneously achieve the best trade-off between coverage and power consumption, (c) The more EDs in the same cluster, the higher the DR allocation should be, (d) Exhaust the distance limits of each DR cluster in order to use as many DRs as possible, while the DR5 cluster should be configured to around 64% of the total EDs [32];
6. Limit the distance-based clusters to equalize as much as possible the maintenance intervals, or distribute the final clusters according to power needs to determine which power capacity configuration should be used in each case (if this is a possible capability for the deployment).

## 5. Conclusions & Future Work

In this study we have extensively summarized common issues that affect communication performance in LoRaWAN networks addressed in the literature. Based on this summary we conducted simulation experiments in order to break down widespread issues into crucial factors. We summarized our findings and proposed some safe pathways to deploy a LoRaWAN network and proposed a network design process which is based not only on the network constraints but also in application ones.

In general, clustering algorithms that improve LoRaWAN communication performance is an open issue. Several studies are proposing novel approaches based on neural networks and machine learning techniques [33,34,35,36]. We plan to extend our work on clustering nodes of a LoRaWAN network by applying our findings within a clustering algorithm, so we test it in real networks. We believe that a methodology which will be able to estimate the required number of gateways in an area we want to cover, along with their optimal position, can enable scalable network deployments with efficient budget allocation.

In addition, we believe that extensive improvements to the LoRaWAN deployment process are expected once our approach is facilitated along with recent published methodologies on planning and deployment that take into account cost and security constraints [24].

Finally, in order to further facilitate the network design and deployment process, we are working on an adaptive end system for range testing. This tool will be suitable for the evaluation of LoRaWAN coverage on various terrains, for the estimation of the number of gateways in an area that we want to cover, as well as their position.

## Figures and Tables

**Table 1:** Crucial factors to control and observe.

Factors	Cruciality	Levels
Number of EDs	Has an indirect significant impact on PDR and determines how scalable the network is.	10–6000
Number of GWs	In most cases more GWs cause the whole network to operate differently by impacting the overall performance.	1–8
Distance from ED to GW	By combining distance measurements with connectivity metrics such as RSSI and SNR can determine the effective range for each GW and thus the coverage of the network.	50 m–10 km
Payload size	Payload size directly affects ToA which has an impact in duty cycle compliance.	10–50 bytes
Transmission interval	An important factor which indirectly related with the overall performance of the network and finally the kind of supported applications.	10–72,000 s
Number of retransmissions	Power consumption and scalability can be affected by this ED parameter.	0–8
ACKs	Referred to the percentage of EDs that ask for ACKs and it is a QoS oriented factor that can affect the whole network if not used wisely.	On/Off
TX	Transmitting power can help achieve greater range but power consumption remains an important factor for LoRaWAN adoption.	0–14 dBm
DR	The key feature of LoRaWAN that is mainly controlled by ADR and affects the overall network performance.	DR0–DR5

## Data Availability

Data presented in the paper are available on request from the corresponding author S.P.
